# Food for Brain and Nerves

**Published:** 1884-06

**Authors:** 


### FOOD FOR BRAIN AND NERVES.

The need of special foods to restore the waste caused by excessive use of the nerves has been long recognized. Scientists say that the materials to do this must come from the animal or vegetable kingdom. Vitalized Phos-phites, devised by one of the most eminent physicians of this city, whose merits as a chemist have been recognized by the American Medical Association with several gold medals, are undoubtedly of the highest value. The Phos-phites are truly Brain Foods scientifically prepared. They are made from the wheat germ and the ox brain. The ordinary food contains elements that build up the muscles; but people who write, think, worry, watch by bedsides, study much, have much responsibility, etc., waste nerves, and not muscles. Of all the special foods F. Crosby Co.'s Vitalized Phosphites are the best yet devised by scientific research. The editor has examined many letters from people in various parts of the United States, and they speak of a benefit that no medicine could give. He believes in them, uses them, and will bear testimony at any time to their efficacy for the purposes claimed by the inventor.

E. L. Kellogg,

Editor of *School Journal.*

